# Progesterone interferes with microtubule-dependent cell division in *Toxoplasma gondii*

**DOI:** 10.1186/s13071-026-07442-w

**Published:** 2026-07-09

**Authors:** Zhili Shan, Zifu Zhu, Na Yang, Yanqun Pei, Jing Liu, Yong Fu, Qun Liu

**Affiliations:** 1 State Key Laboratory of Veterinary Public Health and Safety, College of Veterinary Medicine, China Agricultural University, No.2 Yuanmingyuan West Road, Beijing, 100193 China; 2 Key Laboratory of Animal Epidemiology of the Ministry of Agriculture and Rural Affairs, College of Veterinary Medicine, China Agricultural University, Beijing, 100193 China; 3 National Animal Protozoa Laboratory, College of Veterinary Medicine, China Agricultural University, Beijing, 100193 China

**Keywords:** *Toxoplasma gondii*, Progesterone, Microtubule-associated pathways, Centrosome division

## Abstract

**Background:**

*Toxoplasma gondii* is an opportunistic intracellular parasite that can cause severe reproductive disorders during pregnancy. Progesterone is markedly elevated during pregnancy and has been shown to affect the replication of T. gondii. However, the downstream cellular processes and molecular mechanisms underlying progesterone-mediated regulation of parasite replication remain unclear.

**Methods:**

Transcriptomic analysis was performed to investigate the global gene expression changes in tachyzoites after progesterone treatment. Differentially expressed genes were subjected to functional enrichment analysis. The effects of progesterone on parasite division were further assessed by immunofluorescence assays targeting subpellicular microtubules, centrosome, and organelles.

**Results:**

Transcriptomic analysis identified 329 differentially expressed genes after progesterone treatment, which were mainly enriched in microtubule-associated pathways, including microtubule motor activity and microtubule-based movement. Although the overall structure of subpellicular microtubules showed no detectable alteration, progesterone selectively disrupted division of the outer core of the centrosome, while the inner core of the centrosome was largely unaffected. Further analysis of organelle division showed that progesterone mainly interfered with early events of endodyogeny, including the segregation of the centrosome, Golgi, and apicoplast, whereas later division-related structures were less affected.

**Conclusions:**

These findings indicate that progesterone impairs T. gondii replication by selectively interfering with microtubule-dependent processes, especially outer core centrosome division. This study provides new insight into how the pregnancy-associated hormone progesterone regulates parasite replication.

**Graphical Abstract:**

**Figure:**
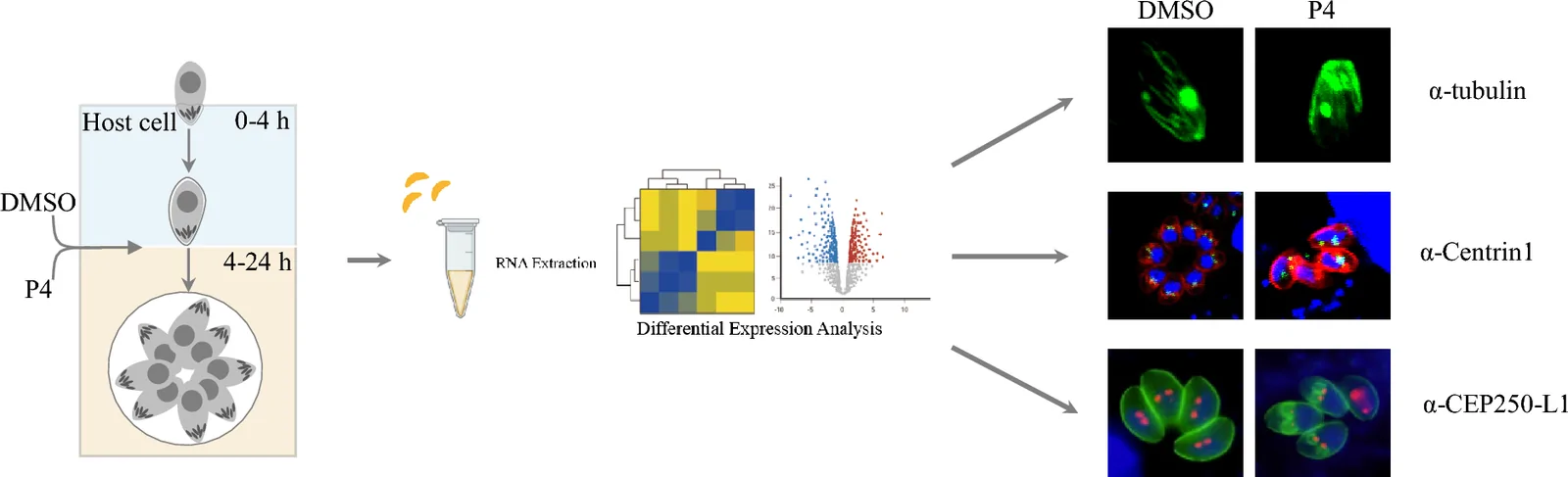

**Supplementary Information:**

The online version contains supplementary material available at https://doi.org/10.1186/s13071-026-07442-w.

## Background

*Toxoplasma gondii* is a highly successful parasite that infects almost all warm-blooded animals, including humans. It is an opportunistic pathogen. In particular, pregnancy in both humans and animals increases the susceptibility to infection, which can lead to miscarriage and stillbirth due to the specific physiological changes that occur during pregnancy [1]. One of the most notable changes is the dramatic increase in progesterone levels compared with non-pregnant states [2, 3].

While sex hormones are well known to modulate host immune responses. Increasing evidence also suggests that they can directly influence parasite physiology, affecting proliferation and developmental progression. For example, progesterone and estradiol have been reported to alter replication in several pathogens, such as *Plasmodium falciparum* [4] and *Taenia crassiceps* [5]. However, the molecular mechanisms underlying these hormonal effects remain poorly understood.

Our previous study demonstrated that progesterone inhibits the invasion and replication of *T. gondii* and identified TgPGRMC as a mediator of this effect. However, the downstream cellular processes underlying progesterone-mediated regulation of parasite replication remain poorly understood [6]. Replication of *T. gondii* occurs through a specialized form of internal budding termed endodyogeny, in which two daughter cells are assembled within the mother cell [7, 8]. The highly coordinated process requires the duplication and segregation of organelles throughout the cell cycle. The sequence begins with centrosome division, followed by division of the Golgi, apicoplast, nucleus, cytoskeleton, endoplasmic reticulum (ER) and finally the mitochondrion [9]. The centrosome is one of the two sites of microtubule-organizing centers (MTOC) in *T. gondii* and serves as the primary site for spindle assembly [10, 11]. It exhibits a bipartite organization, with the inner core controlling the nuclear cycle and spindle formation and the outer core governing daughter cell budding and cytoskeletal assembly [12, 13]. This structural and functional segregation enables the independent yet coordinated regulation of nuclear division and cytokinesis-like processes. A second MTOC is located at the apical polar ring (APR), which organizes the subpellicular microtubules (SPMTs) forming a scaffold that maintains parasite shape [14–16].

Microtubule-associated proteins (MAPs), including motor proteins such as kinesin and dynein, are essential for organizing microtubule networks and maintaining centrosome function [17]. Dynein mediates centrosome attachment and migration along the nuclear envelope during interphase and prophase, and helps maintain its association with the spindle poles [18]. Kinesin Eg5 drives centrosome separation in late G2 and early prophase [19]. Cortical dynein generates centering forces by pulling the centrosome toward the cell center, while microtubule polymerization exerts outward pressure on the cortex, together contributing to precise centrosome positioning [18, 20, 21]. Together, these factors ensure proper microtubule architecture, centrosome positioning and faithful segregation of cellular contents.

In this study, we combined transcriptomic profiling to investigate the effects of progesterone on parasite division. Our findings show that progesterone predominantly alters the transcription of microtubule-associated genes and selectively perturbs outer core of centrosome and early organelle duplication. These findings provide evidence for how progesterone regulates parasite division.

## Methods

### Culture and collection of *T. gondii*

Parasites (RHΔ*ku80* strain) were grown in vitro on HFF cells (cell line CRL-1634; American Type Culture Collection [ATCC], Manassas, VA, USA) and serially passaged in Dulbecco's modified Eagle medium (DMEM; Macgene Biotechnology, Beijing, China; Cat. no. CM10013) supplemented with 2% fetal bovine serum (Macgene Biotechnology; Cat. no. CS002) at 37 °C with 5% CO_2_. The culture medium was changed 24 h after inoculation. When the parasites were ready to egress, they were scraped off the cell and agitated 3-5 times in 5-ml disposable sterile syringes, then passed through a 5-µm sterile filter to remove the cell debris. *Toxoplasma gondii* was finally collected after centrifugation at 2800 rpm for 10 min.

### Generation of transgenic *T. gondii* strains

To produce hemagglutinin (HA)-tagged TgAPR1 (TGGT1_315510), TgIMC29 (TGGT1_243200), TgCEP250-L1 (TGGT1_290620) and TgSFA2 (TGGT1_205670) parasite lines, a DNA fragment was amplified using the pLIC-HA-DHFR plasmid as a template. The PCR product and the pSAG1::Cas9-U6 plasmid were co-transfected into the RH∆*ku80* strain, and positive strains were selected with pyrimethamine. Recombinant strains were identified using an immunofluorescence assay (IFA). The sequences of all primers used in this study are listed in Additional file 1: Table S1.

### Transcriptomic sequencing and data processing

Total RNA was extracted using the TRI reagent (Sigma-Aldrich, St. Louis, MO, USA; cat no. T9424) from the two groups of parasites with three biological replicates each. The quality of the RNA samples was assessed by measuring the A260/A280 absorbance ratio (OD260/280) using a Nanodrop ND-2000 system (Thermo Fisher Scientific, Waltham, MAUSA) and RNA integrity (RIN) values using an Agilent Bioanalyzer 4150 system (Agilent Technologies, Santa Clara, CA, USA). High-quality RNA samples with OD260/280 ranging from 1.8 to 2.2 and RIN values ≥ 6.5 were used for construction of the complementary DNA (cDNA) using the ABclonal mRNA-seq Lib Prep Kit (ABclonal Biotechnology, Wuhan, China; Cat. no. RK20349) following the manufacturer’s instructions. Sequencing was performed on an Illumina Novaseq 6000/MGISEQ-T7 instrument (Illumina Inc., San Diego, CA, USA). After sequencing, all analyses were performed using an in-house pipeline provided by Shanghai Applied Protein Technology (Shanghai, China). Briefly, the raw data in FASTQ format were first processed to remove the adapter sequence and low-quality reads using FASTQ software (v 0.19.5). The clean reads were separately aligned to the reference genome of *T. gondii* using HISAT2 software (v 2.0.5) to obtain mapped reads. Feature Counts (v 1.5.0-p3) was used to count the read numbers mapped to each gene, and then normalized with the FPKM (fragments per kilobase of exon per million mapped fragments) method. Differential expression analysis was performed using the DESeq2 method. Differentially expressed genes (DEGs) with |log2FC|> 1 and *P*-adjusted (*P*-adj) < 0.05 were considered to be significantly expressed. Functional enrichment analysis of Gene Ontology (GO) terms was conducted using the “clusterProfiler” package in R software ® Foundation for Statistical Computing, Vienna, Austria), and the GO function was considered to be significantly enriched when *P*-adj < 0.05.

### Quantitative PCR analysis of microtubule-associated genes

Total RNA from progesterone-treated and untreated parasites was extracted using TRI reagent (Sigma-Aldrich; Cat. no. T9424). Microtubule-associated genes showing differential expression in the transcriptomic analysis were selected for validation by quantitative PCR (qPCR). The actin (ACT1) (TGGT1_209030) was used as the reference gene. cDNA was synthesized from total RNA using a reverse transcription kit (AGbio China, Changsha, Hunan, China; Cat. no. AG11705). The qPCR assays were performed using the One Step RT-qPCR SYBR Green Kit (Vazyme Biotech, Nanjing, China; Cat. no. Q226-01). Amplification was carried out on a LightCycler 480 system (Roche, Basel, Switzerland).

### Deoxycholate extraction

Deoxycholate (DOC) extraction was performed as described previously by Wu et al. [22], with minor modifications. RH∆*ku80* cells were infected and cultured for 4 h, followed by treatment with progesterone or DMSO for an additional 20 h. Freshly released tachyzoites were collected and dropped onto lysine-coated cell slides, then incubated at 37 ℃ for 30 min. The slides were treated with 5 mM DOC (Yeasen Biotechnology, Shanghai, China; Cat. no. 60334ES10) at room temperature for 20 min. Anti-acetylated tubulin (1:100, gift from Prof. Shaojun Long, Sun Yat-Sen university, School of Public Health, Shenzhen) and anti-TgIMC1 were used to perform the immunofluorescence assay as described previously [22].

### Immunofluorescence assay

All IFAs were performed following a similar protocol. HFF cells were infected with parasites, then fixed with 4% paraformaldehyde, permeabilized with 0.25% TritonX-100 and blocked with 3% bovine serum albumin for 1 h at room temperature. Subsequently, coverslips were incubated with primary antibodies and followed by FITC- or Cy3-conjugated secondary antibodies (1:100; Proteintech, Shanghai, China; Cat. no. SA00003-2 and SA00009-2, respectively) and DAPI (1:100, Solarbio, Beijing, China; Cat. no. ID22502). The primary antibodies used included anti-H3K4me3 (PTM Bio, Shanghai, China; Cat. no. PTM-613), anti-HA, anti-TgIMC1, anti-TgGAP45, anti-TgACP, anti-TgBIP and anti-TgCentrin1, anti-TgHSP60 and anti-α-Tubulin (a gift from Prof. Shaojun Long) at the following dilutions: 1:100, 1:50, 1:300, 1:300, 1:500, 1:500, 1:500, 1:500, 1:200, respectively. The parasites were then visualized using a Nikon Ni-E microscope (C2 plus; Nikon Corp., Tokyo, Japan) equipped with a DS-Ri2 microscope camera, and analyzed using the Ni-E software system installed in the NIS-Elements AR package (Nikon Corp.).

### Organelle labeling

Intracellular parasites were labeled with anti-TgCentrin1, anti-TgACP, anti-TgBIP, anti-TgHSP60 and anti-H3K4me3 to label the centrosome, apicoplast, ER, mitochondria and nucleus, respectively.

The cytoskeleton was labeled with anti-α-Tubulin. The first culture time for cytoskeleton labeling was the same as previously described. The second culture period for cytoskeletal labeling avoided multiples of 6-8 h to minimize interference with the cell cycle. The cells were then fixed and both intracellular and extracellular IFAs were performed.

### Statistical analysis

GraphPad Prism software (v 10.1.2; GraphPad Software Inc., San Diego, CA, USA) was used for plotting data and statistical analysis. Means are shown by graphs, while standard errors of the means (SEMs) are represented by error bars. A t-test was performed on the data to determine the differences between the groups. Regarding the results, *P* > 0.05 (not significant [ns]) indicates that there is no significant difference; *P < 0.05 suggests a statistically significant difference; **P < 0.01 denotes a very significant difference; and ***P < 0.001 suggests a highly significant difference.

## Results

### Progesterone treatment modulates the transcription of microtubule-associated genes

**Fig. 1:**
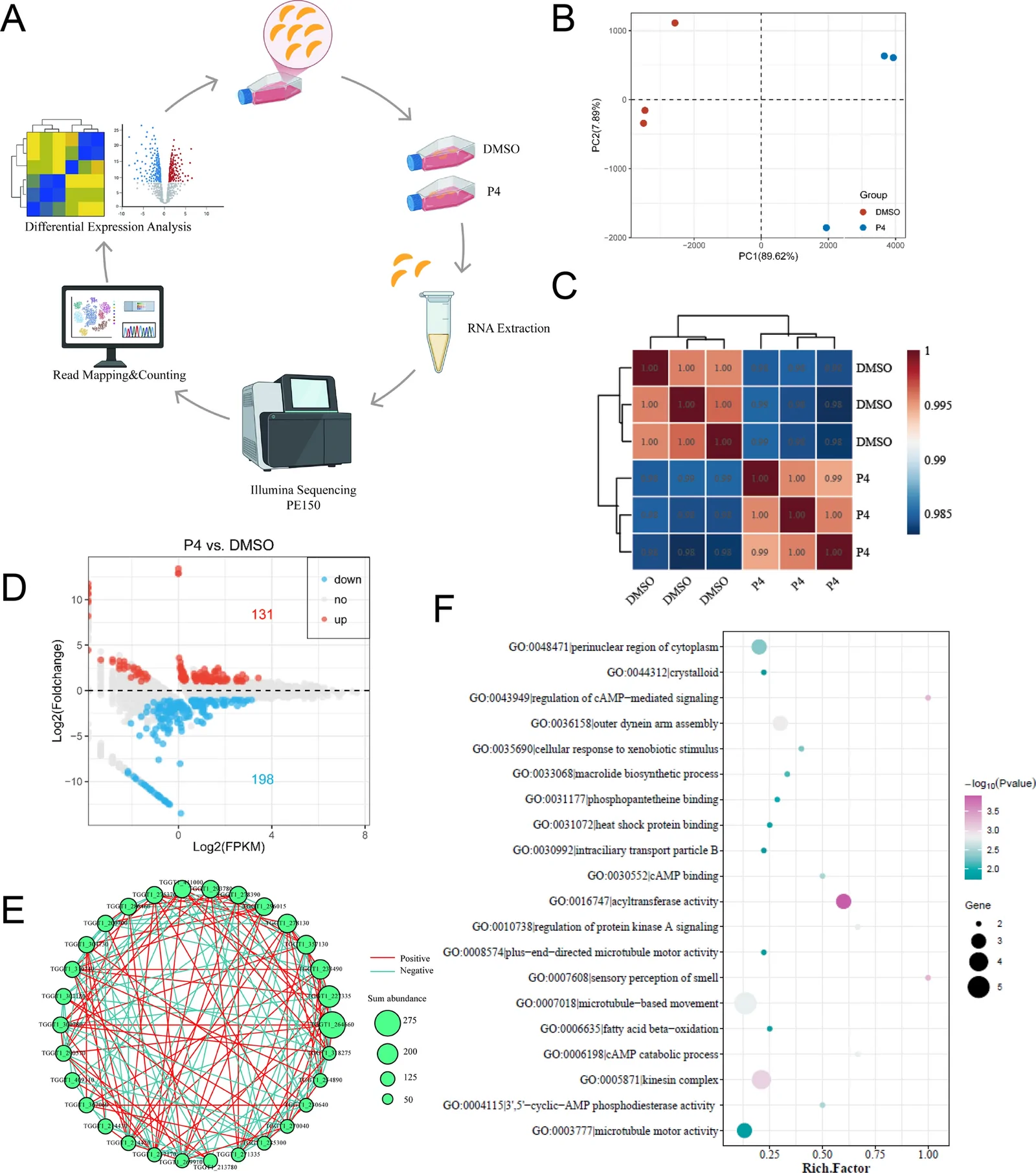
Transcriptomic analysis of *Toxoplasma gondii* in response to progesterone treatment. **A** Schematic overview of the experimental design and transcriptomic workflow used to analyze *T. gondii* treated with (P4) or without progesterone (DMSO). **B** Principal component analysis (PCA) of RNA-sequencing samples showing the distribution of biological replicates from control and progesterone-treated groups. **C** Heatmap showing Pearson correlation coefficients among different transcriptome samples. **D** Volcano plot showing the differentially expressed genes (DEGs) identified between progesterone-treated and control parasites. **E** Correlation network constructed based on the identified DEGs. **F** GO enrichment analysis of DEGs identified from the comparison between the two groups. DMSO, Dimethyl sulfoxide; GO, Gene Ontology

Previous results indicated that progesterone affects the replication of *T. gondii*. To investigate the molecular basis of this effect, we designed a workflow combining transcriptomic analysis of parasites treated with or without progesterone (P4) (Fig. 1a).

We performed transcriptomic analysis to gain more insights into the global transcriptional responses under the treatment regimen. After quality control filtering of high-throughput sequencing, a total of 40G clean reads form six 6 RNA-sequencing (RNA-seq) libraries were obtained with an average of 6.45G clean data sequenced per library, of which an average of 92.91% were mapped onto the reference genome of *T. gondii*. First, principal component analysis (PCA) showed the consistency between the three biological replicates with high correlation in same group (Fig. 1b), which were divided by PC1 (89.62%) and PC2 (7.89%). Consistent with the PCA results, sample correlation analysis further confirmed the high similarity among the biological replicates within the same group and the distinct transcriptional profiles between the progesterone-treated (P4) and control groups (DMSO) (Fig. 1c). Moreover, a total of 329 DEGs, including 131 upregulated and 198 downregulated genes, were identified by the differential expressed analysis, as indicated by the colored points in the volcano plot (Fig. 1d); of these, 126 genes were annotated as coding proteins and 203 genes were classified as hypothetical proteins. To explore the potential relationships among these DEGs, we constructed a correlation network. This network revealed that DEGs were tightly connected, suggesting that progesterone alters the coordination expression of these genes (Fig. 1e). To further link these patterns to biological function, we aligned the identified DEGs by comparing these to the GO database. Notably, the DEGs were predominantly enriched in microtubule-associated pathways, including outer dynein arm assembly (GO:0036158), microtubule motor activity (GO:0003777), kinesin complex (GO:0005871) and microtubule-based movement (GO:0007018) (Fig. 1f).

### Subpellicular microtubules are not affected by progesterone treatment

**Fig. 2:**
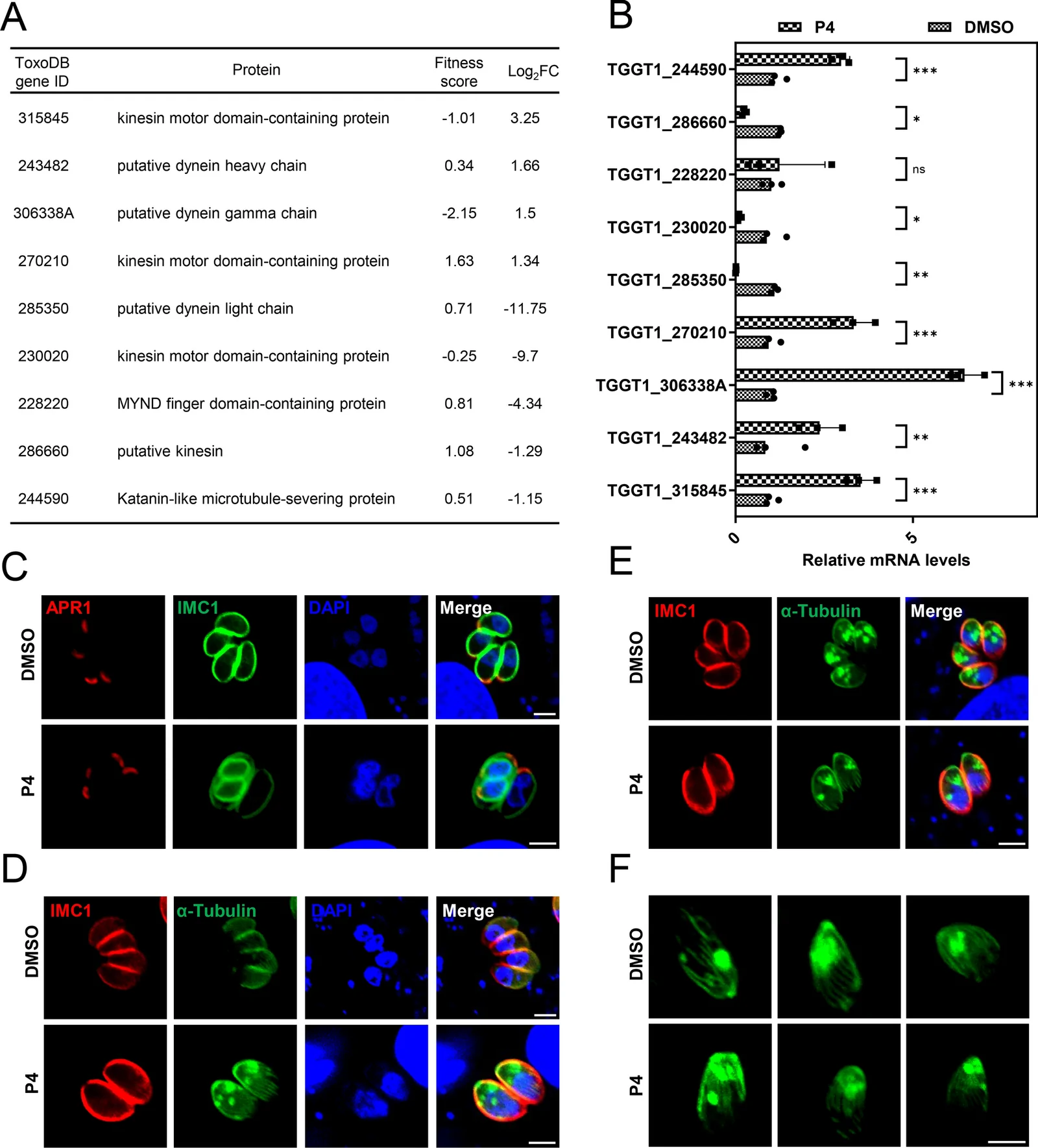
Progesterone altered the transcription of motor proteins but not of subpellicular microtubule organization. **A** Table showing kinesin- and dynein-related genes identified as differentially expressed in the transcriptomic analysis. **B** Quantitative PCR validation of selected genes from the transcriptomic dataset. Parasites were allowed to replicate for 4 h, followed by treatment with 1 μM progesterone for 20 h prior to RNA extraction. Data represent three biological replicates, each with three technical replicates. **C** Immunofluorescence assay (IFA) showing the localization of TgAPR1 in control (DMSO) and progesterone-treated (P4) parasites using mouse anti-HA (TgAPR1, red) and rabbit anti-TgIMC1 (green). Scale bar: 2 µm. **D** IFA of intracellular parasites cultured for 24 h showing tubulin distribution, using mouse anti-TgIMC1 (red) and rabbit anti-α-tubulin (green). Scale bar: 2 µm. **E** IFA of parasites at different incubation time points showing tubulin organization in control (DMSO) and progesterone-treated (P4) groups. Scale bar: 2 µm. **F** Deoxycholate extraction assay assessing the organization of subpellicular microtubules. Scale bar: 2 µm. Asterisks in** B** indicate a significant difference at *P < 0.05, **P < 0.01 and ***P < 0.001; ns, no significant difference. DMSO, Dimethyl sulfoxide

Transcriptomic analysis revealed that numerous genes encoding microtubule-associated motor proteins, including kinesin and dynein, were differentially expressed upon progesterone treatment. Although the specific functions of these proteins in *T. gondii* remain largely uncharacterized, they are well known in other eukaryotes to participate in microtubule organization, spindle assembly and intracellular transport. To validate the transcriptomic data, we selected these microtubule-associated genes for qPCR analysis (Fig. 2a). The qPCR results largely confirmed the changes in transcription levels observed in the transcriptomic analysis (Fig. 2b). Given the large number of kinesin and dynein and the limited understanding of their specific functions in *T. gondii*, we next examined microtubule structure to assess their potential roles in microtubule organization and function.

To investigate whether progesterone affects the non-centrosomal microtubule network associated with the APR, which organizes subpellicular microtubules, we first examined the APR using TgAPR1 as a marker. The results showed that there were no detectable differences in TgAPR1 localization between control (DMSO) and progesterone-treated (P4) parasites (Fig. 2c).

Subpellicular microtubules were examined by IFA using anti-α-tubulin. After 20 h of progesterone treatment, tubulin was observed to aggregate near the apical side of the nucleus (Fig. 2d). Further experiments adjusting the duration of parasite culture revealed that similar tubulin accumulation also occurred in control parasites, indicating that this phenomenon is dependent on incubation time rather than being a specific effect of progesterone (Fig. 2e). Also, DOC extraction assays revealed no detectable disorganization of the subpellicular microtubules (Fig. 2f). Taken together, these observations suggest that progesterone does not markedly affect the non-centrosomal microtubule network.

### Progesterone selectively impairs outer core of centrosome division

Although subpellicular microtubules appeared to be largely unaffected by progesterone treatment, the enrichment of microtubule-associated motor proteins among the DEGs suggested that specific microtubule-dependent processes might be impacted. Many kinesins and dyneins are known in other eukaryotes to participate in centrosome division. In *T. gondii*, the centrosome functions as a MTOC that coordinates nuclear division and daughter cell budding. We therefore examined whether progesterone affects the centrosome division in *T. gondii*.

**Fig. 3:**
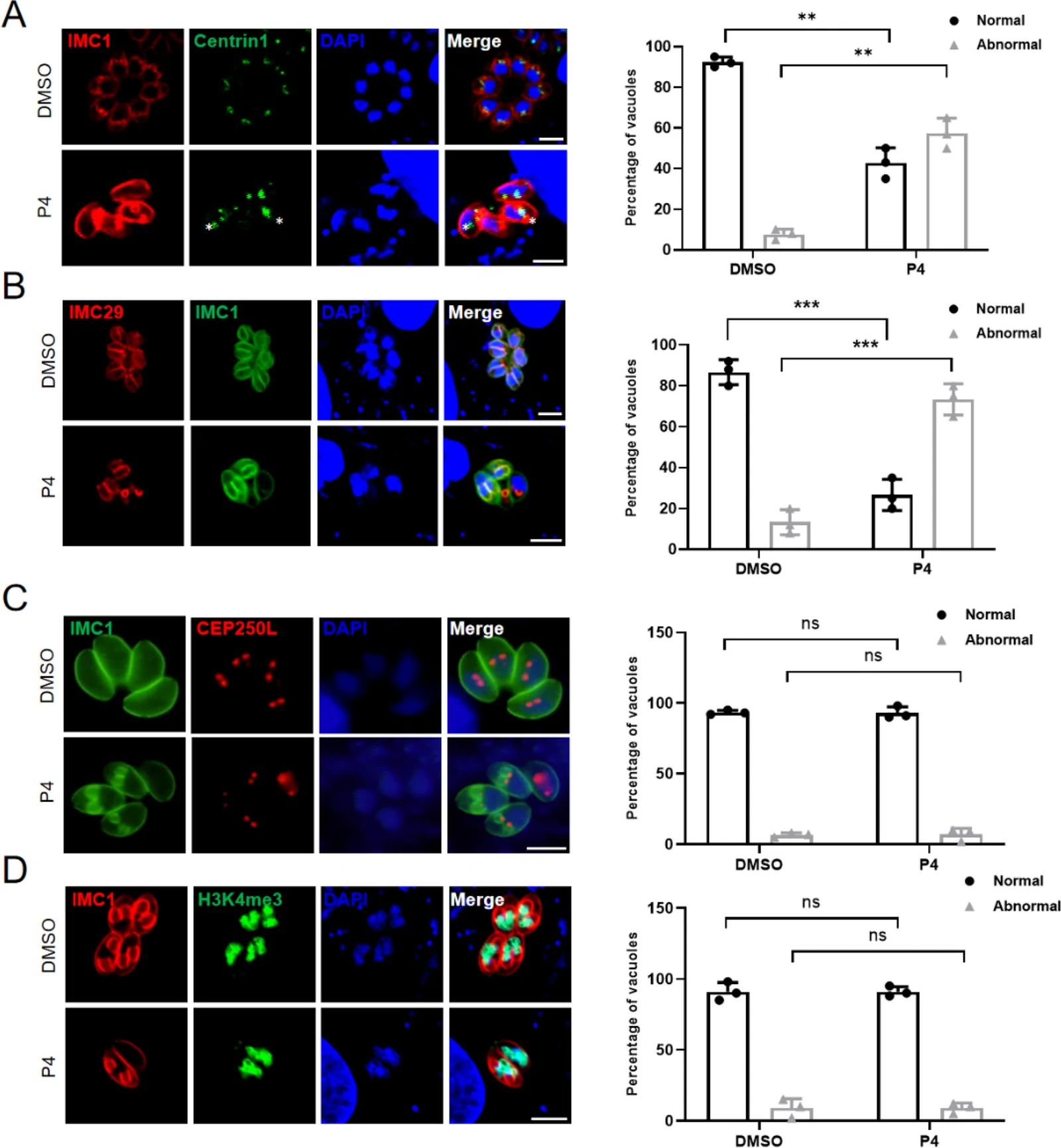
Progesterone disrupts outer core of centrosome division in *Toxoplasma gondii*. **A**, **B** Immunofluorescence assay (IFA) of the outer core and inner membrane complex (IMC) in control (DMSO) and progesterone-treated (P4) parasites using rabbit anti-TgCentrin1 (green) and mouse anti-TgIMC1 (red); mouse anti-HA (IMC29, red) and rabbit anti-TgIMC1 (green). Scale bar: 5 µm. Quantification of vacuoles with normal outer core duplication and IMC budding is shown on the right. **C**, **D** IFA of inner core and nuclear division using mouse anti-HA (TgCEP250-L1, red) and rabbit anti-H3K4me3 (green). Scale bar: 5 µm. Quantification of vacuoles with inner core and nuclear division is shown on the right. Data are the mean ± standard deviation pooled from 3 biological replicates (*N* = 3, *n* = 3). Asterisks indicate a significant difference at **P < 0.01 and ***P < 0.001; ns, no significant difference. DMSO, Dimethyl sulfoxide

The *T. gondii* centrosome consists of two functional cores that coordinate nuclear division and daughter budding, with the inner core controlling the nuclear cycle and the outer core controlling the budding cycle [13, 23]. To monitor outer core division and daughter budding, we performed IFA using TgCentrin1 and TgIMC1, respectively, as markers. Under control conditions, division of the outer core and inner membrane complex (IMC) budding proceeded normally. Upon progesterone treatment, however, we observed disorganized duplication of the outer core of centrosome. Parasitophorous vacuoles (PVs) displayed supernumerary centrosomes (Fig. 3a). Quantitative analysis confirmed a significant increase in outer core duplication defects compared with the controls. These abnormalities were further confirmed using an additional outer core marker, TgSFA2 (data not show). In addition, TgIMC1 and TgIMC29 staining revealed asynchronous IMC budding, and quantitative analysis showed a significant increase in IMC defects compared with control parasites (Fig. 3b). In contrast, the inner core TgCep250-L1 and nuclear division appeared to be unaffected (Fig. 3c, d) [13].

Together, these results indicate that progesterone selectively perturbs outer core of centrosome division and daughter cell budding, while inner core of centrosome division and nuclear division is unaffected.

### Progesterone disrupts the segregation of early dividing organelles

**Fig. 4:**
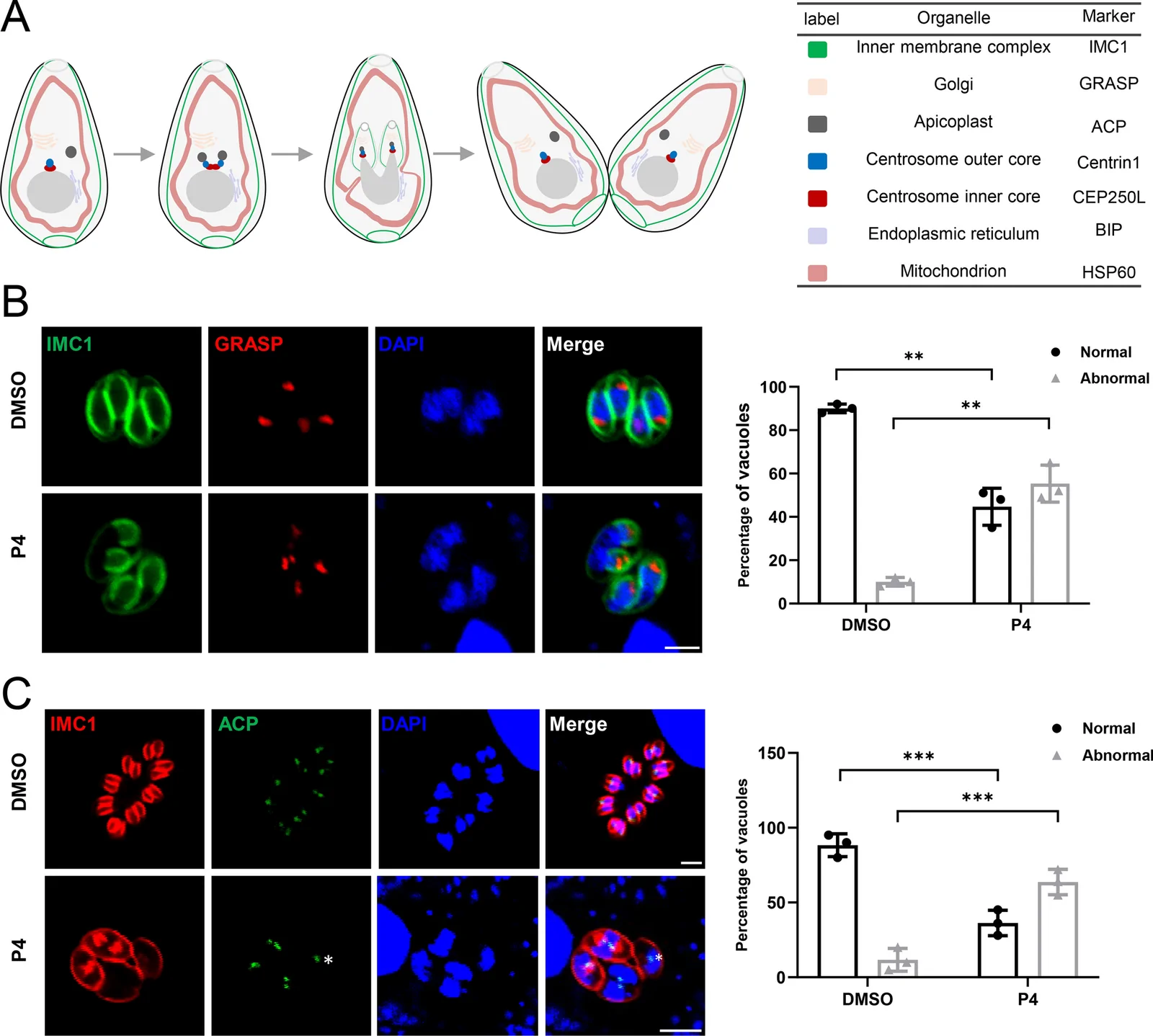
Progesterone disrupts Golgi division and apicoplast segregation in *Toxoplasma gondii*. **A** Schematic of the temporal sequence of organelle duplication during the parasite cell cycle, beginning with centrosome replication, followed by duplication of the Golgi, apicoplast, nucleus, endoplasmic reticulum and mitochondrion. **B** Immunofluorescence assay (IFA) of Golgi division using rabbit anti-TgGRASP (red) in control (DMSO) and progesterone -treated (P4) parasites. Scale bar: 2 µm. Quantification of vacuoles with normal or supernumerary Golgi is shown on the right. **C** IFA of apicoplast segregation using rabbit anti-TgACP (green) in control (DMSO) and progesterone-treated (P4) parasites. Scale bar: 5 µm. Data are the mean ± standard deviation pooled from 3 biological replicates (*N* = 3, *n* = 3). Asterisk indicate a significant difference at **P < 0.01 and ***P < 0.001. DMSO, Dimethyl sulfoxide

Given that progesterone disrupted outer core of centrosome duplication, we next examined whether organelles downstream of centrosome duplication were affected. *Toxoplasma gondii* undergoes a highly ordered sequence of organelle duplication during the cell cycle, beginning with centrosome duplication, followed by the duplication of various organelles, including the Golgi, apicoplast, nucleus, ER and, finally, the mitochondrion (Fig. 4a) [9, 24].

We first assessed Golgi division using TgGRASP as a marker. Under control conditions, each daughter parasite inherited a single Golgi apparatus. Following progesterone treatment, 55% of parasites exhibited supernumerary Golgi (Fig. 4b). Next, apicoplast segregation was examined using TgACP staining. Progesterone-treated parasites showed a significant increase in the absence of a detectable apicoplast, with 64% of vacuoles lacking apicoplasts compared with 11% in control parasites (Fig. 4c).

### Late-dividing organelles are unaffected by progesterone

**Fig. 5:**
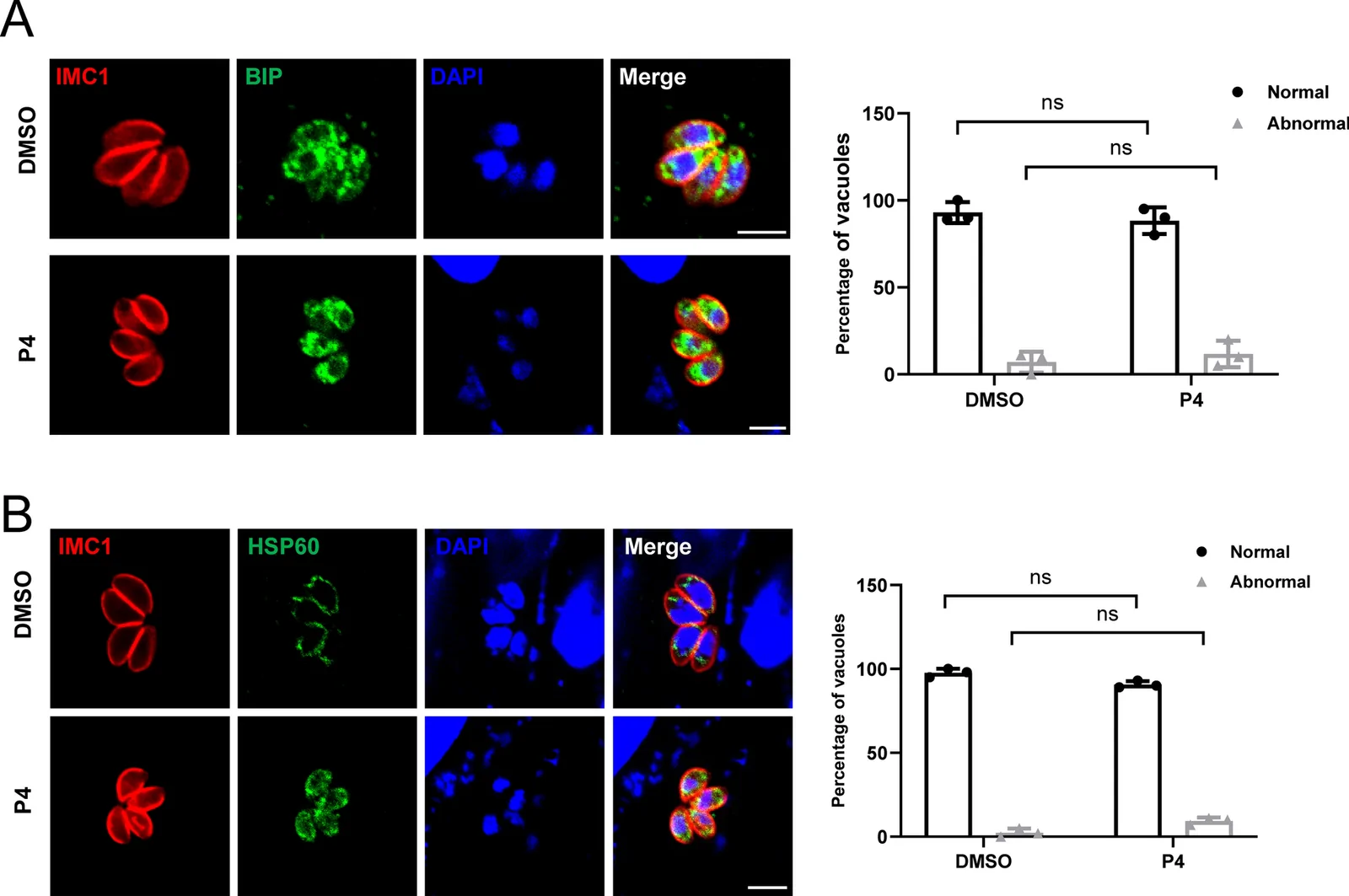
Progesterone does not affect endoplasmic reticulum (ER) and mitochondrial morphology. **A** Immunofluorescence assays (IFA) of the ER using rabbit anti-TgBiP (green) in control (DMSO) and progesterone-treated (P4) parasites. Scale bar: 5 µm. **B** IFA of mitochondria using rabbit anti-TgHSP60 (green) in control (DMSO) and progesterone-treated (P4) parasites. Scale bar: 5 µm. Data are the mean ± standard deviation pooled from 3 biological replicates (*N* = 3, *n* = 3). DMSO, Dimethyl sulfoxide; ns, no significant difference

To assess the effects of progesterone on other organelles, we examined the ER and mitochondria using specific markers. By staining TgBiP for the ER, we observed that the overall ER network remained intact in progesterone-treated parasites, with no apparent fragmentation or mis-localization. Similarly, staining with a mitochondrial marker revealed that the majority of parasites maintained a continuous mitochondrial network (Fig. 5a, b).

In contrast to the Golgi and apicoplast, which displayed clear defects in segregation under progesterone treatment, the ER and mitochondria maintained normal structure. These observations suggest that progesterone treatment predominantly impaired early dividing organelles, whereas the ER and mitochondria appeared to be unaffected.

## Discussion

Pregnant hosts exhibit increased susceptibility to *T. gondii* infection. Progesterone is one of the most variable physiological factors during pregnancy, and progesterone levels are significantly raised during pregnancy. In this study, we observed that progesterone selectively disrupts outer core of centrosome duplication and early organelle segregation. The transcriptomic analysis showed that progesterone treatment predominantly alters the transcription of microtubule-associated proteins, providing potential evidence for defects in microtubule-dependent division processes.

In our previous study, progesterone treatment for 3 h was sufficient to induce measurable effects on *T. gondii* [6]. However, hormone-induced transcriptional responses often show temporal patterns in which only a limited number of genes respond at early time points, whereas extensive transcriptome reprogramming emerges at later time points following sustained exposure [25, 26]. For example, in estrogen-treated tissues, significant numbers of genes exhibit differential expression by 6 h after treatment, indicating that extensive transcriptomic changes require prolonged stimuli rather than very short exposure [27]. Therefore, in the present study we significantly prolonged progesterone treatment to capture transcriptional changes in replication-associated genes and allow observation of their effects on parasite replication across multiple consecutive division cycles. This modified approach facilitated the identification of both the immediate and cumulative impacts of progesterone on the replication process.

During intracellular growth, *T. gondii* completes one round of endodyogeny approximately every 6-8 h. By 4 h post-invasion, most parasites are progressing through the cell cycle and approaching the onset of the subsequent budding cycle. Initiating progesterone treatment at this time point corresponds to intervention at a critical window preceding daughter cell formation. Maintaining exposure for 20 h ensured coverage of the full execution phase of cell division and encompassed approximately two to three replication cycles. This experimental approach facilitates identification of replication-associated gene transcription changes and the observation of their compounded effects on parasite replication over two to three successive division cycles, thereby capturing both direct and cumulative impacts on the replication process.

The flexibility of cell division is believed to be controlled by the dual core centrosome, with the inner core controlling the nuclear cycle and the outer core controlling the budding cycle [13]. Surprisingly, using TgCentrin1 and TgSFA2 as markers for the outer core, we found that progesterone caused abnormal division of outer core. Centrosome separation and duplication are driven by molecular motors, particularly kinesin and dynein motors. EG5 is an evolutionary conserved kinesin that is essential for centrosome separation. It creates an outward pushing force between the two centrosomes, promoting centrosome separation [28, 29]. Dynein, on the other hand, exerts inward pulling force essential for centrosome positioning [18]. TgMyoF and TgActin govern the positioning of centrosomes during parasite division, and it has been shown that *T. gondii* may use the dynamical system to control centrosome division [30]. Many of the DEGs identified in our transcriptome analysis were kinesin and dynein, suggesting that progesterone may disrupt the correct division and duplication of the outer core and may cause a severe block to budding by affecting kinesin and dynein.

Early dividing organelles, including the Golgi apparatus and apicoplast, exhibited defects in segregation following progesterone treatment, whereas later dividing organelles, such as the endoplasmic reticulum and mitochondria, remained largely unaffected. These observations indicate that progesterone selectively interferes with specific steps in organelle replication. However, the underlying molecular basis for this selectivity remains unclear.

## Conclusions

Collectively, these findings demonstrate that progesterone affects *T. gondii* division by altering the transcription of microtubule-associated proteins, resulting in defects in outer core of centrosome duplication and daughter cell budding.

## Supplementary Information

**Additional file 1.**

**Additional file 2.**

**Additional file 3.**

## Data Availability

All datasets generated for this study are included in the manuscript files.
