# Supplementary material for: Progesterone interferes with microtubule-dependent cell division in Toxoplasma gondii
Source: Parasit Vectors. 2026 Jul 9;19:428. doi: 10.1186/s13071-026-07442-w (PMC13632415; doi:10.1186/s13071-026-07442-w)
Supplement: Supplementary file 1 [file 13071_2026_7442_MOESM1_ESM.pdf]

Supplementary Figure

Supplementary Figure 1

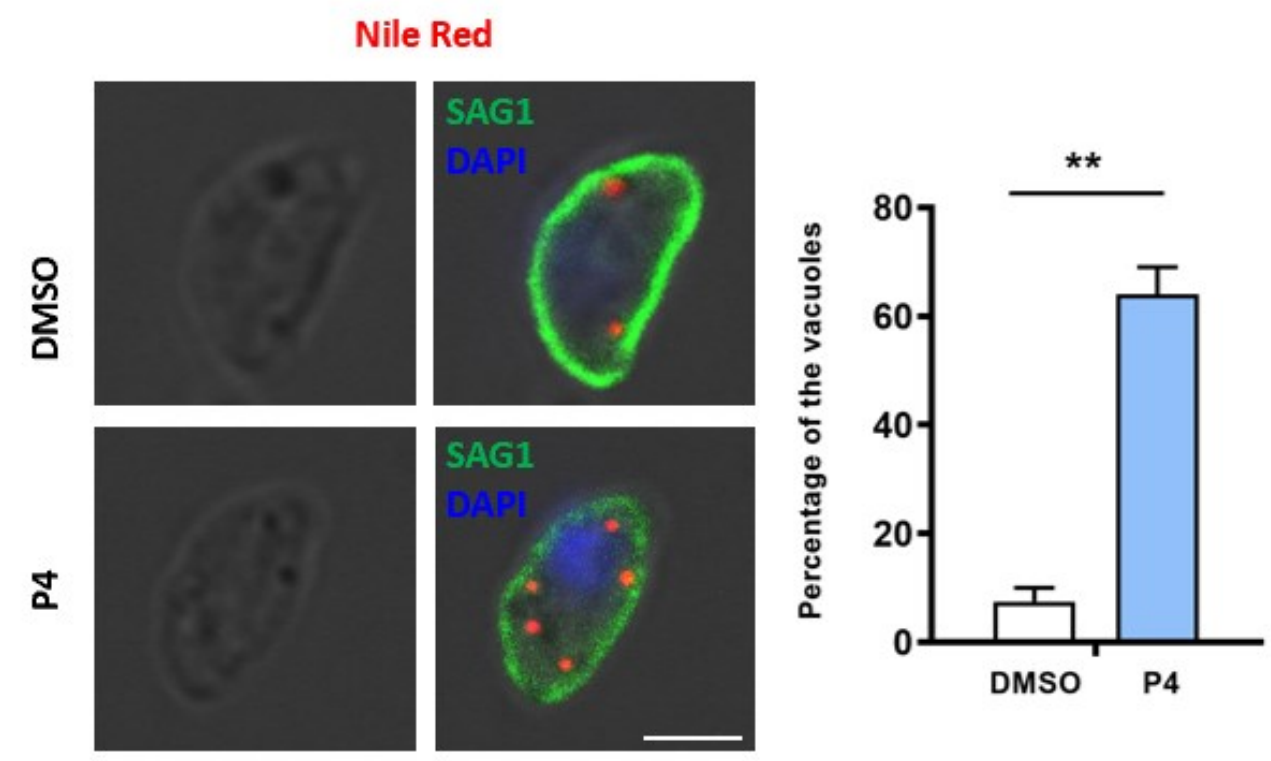

Progesterone treatment increases lipid droplet accumulation in *T. gondii.*

Progesterone-treated and untreated parasites were collected and fixed with 4% paraformaldehyde. Extracellular IFA were performed. The parasite was labeled using anti-TgSAG1, and lipid droplets were stained with Nile Red (1:20 dilution). In the control group, parasites typically contained 1-2 lipid droplets, whereas progesterone-treated parasites showed an increase number of lipid droplets Scale bar: 2 μm.

Supplementary Figure 2

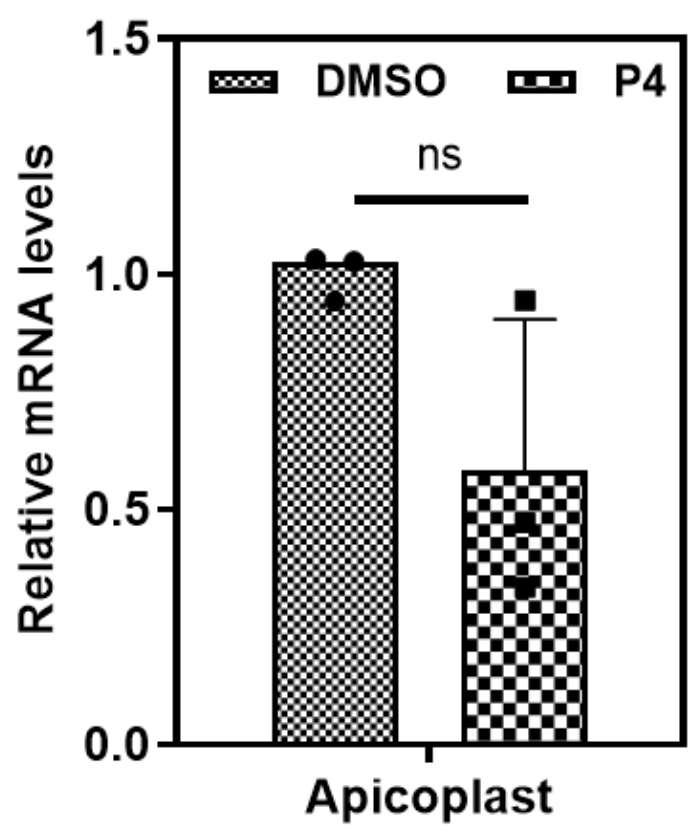

Progesterone does not affect apicoplast DNA abundance.

Relative abundance of apicoplast DNA was quantified by qPCR and normalized to mitochondrial DNA as a reference. No significant difference was observed between progesterone-treated and control groups. The experimental procedures followed previously published methods [1, 2, 3]. Primer sequences used in this study are listed

below: mitochondrial F: CGTAGTAACCTCCAAGTAGCCAAGG, mitochondrial R: AACTACCGCTTGGATGTCTGGTTTAG; apicoplast F: TATGCCCGTACTAAAACTGACACA, apicoplast R: CCTACTTATATGCTTTCAGTAGTTATT

Supplementary Figure 3

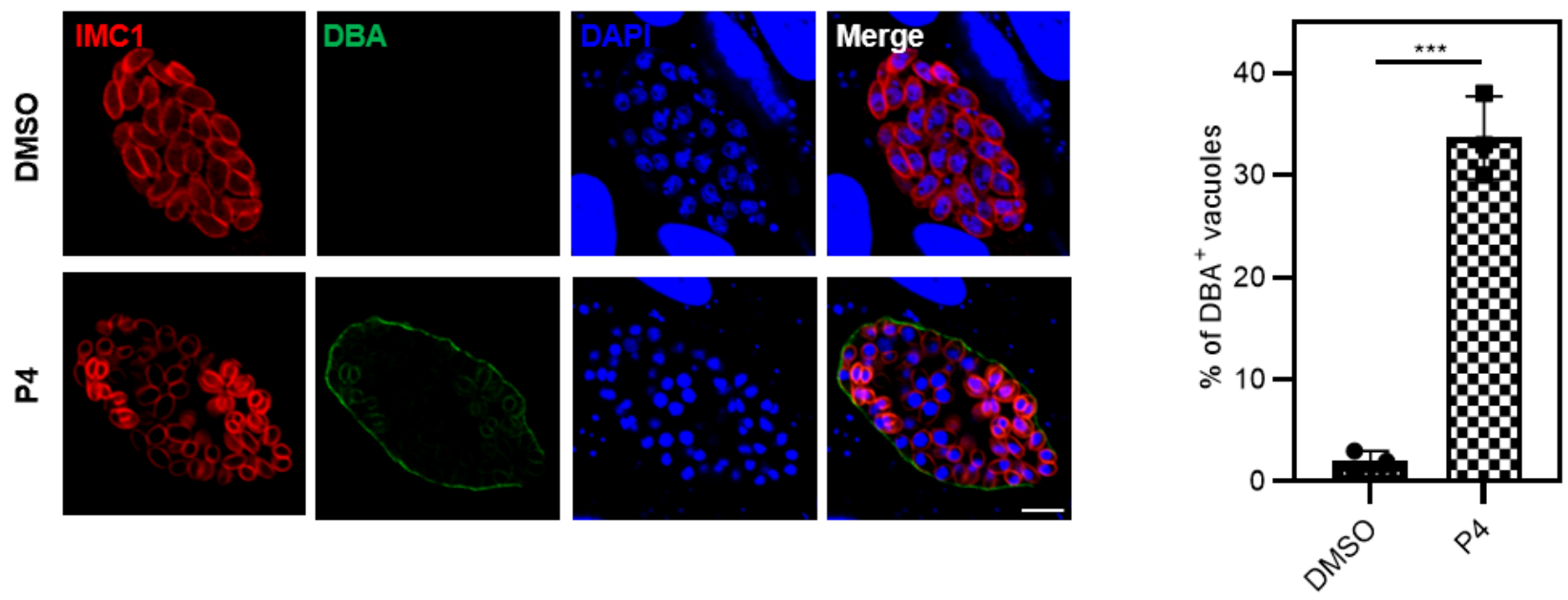

Progesterone enhances DBA-positive vacuoles formation.

Progesterone treatment significantly increased the formation of DBA-positive vacuoles compared with control groups. Quantification of DBA-positive vacuoles is shown in the right panel. Scale bar: 5 µm.

1. Xue Y, Theisen TC, Rastogi S, Ferrel A, Quake SR, Boothroyd JC. A single-parasite transcriptional atlas of Toxoplasma Gondii reveals novel control of antigen expression. eLife. 2020;9; doi: 10.7554/eLife.54129.
2. Martins-Duarte É S, Sheiner L, Reiff SB, de Souza W, Striepen B. Replication and partitioning of the apicoplast genome of Toxoplasma gondii is linked to the cell cycle and requires DNA polymerase and gyrase. International journal for parasitology. 2021;51 6:493-504; doi: 10.1016/j.ijpara.2020.11.004.
3. Garbuz T, Arrizabalaga G. Lack of mitochondrial MutS homolog 1 in Toxoplasma gondii disrupts maintenance and fidelity of mitochondrial DNA and reveals metabolic plasticity. PloS one. 2017;12 11:e0188040; doi: 10.1371/journal.pone.0188040.
